# Saikosaponin D Attenuates Pancreatic Injury Through Suppressing the Apoptosis of Acinar Cell via Modulation of the MAPK Signaling Pathway

**DOI:** 10.3389/fphar.2021.735079

**Published:** 2021-10-21

**Authors:** Caixia Li, Lihua Cui, Lanqiu Zhang, Lei Yang, Yuzhen Zhuo, Jialin Cui, Naiqiang Cui, Shukun Zhang

**Affiliations:** ^1^ Tianjin Key Laboratory of Acute Abdomen Disease Associated Organ Injury and ITCWM Repair, Institute of Acute Abdominal Diseases of Integrated Traditional Chinese and Western Medicine, Tianjin Nankai Hospital, Nankai Clinical College, Tianjin Medical University, Tianjin, China; ^2^ The Clinical Medicine, Tianjin Medical University, Tianjin, China

**Keywords:** chronic pancreatitis, saikosaponin d, AR42J acini cell, apoptosis, MAPK pathway

## Abstract

Chronic pancreatitis (CP) is a progressive fibro-inflammatory syndrome. The damage of acinar cells is the main cause of inflammation and the activation of pancreatic stellate cells (PSCs), which can thereby possibly further aggravate the apoptosis of more acinar cells. Saikosaponind (SSd), a major active ingredient derived from Chinese medicinal herb bupleurum falcatum, which exerted multiple pharmacological effects. However, it is not clear whether SSd protects pancreatic injury of CP via regulating the apoptosis of pancreatic acinar cells. This study systematically investigated the effect of SSd on pancreatic injury of CP *in vivo* and *in vitro*. The results revealed that SSd attenuate pancreatic damage, decrease the apoptosis and suppress the phosphorylation level of MAPK family proteins (JNK1/2, ERK1/2, and p38 MAPK) significantly in the pancreas of CP rats. In addition, SSd markedly reduced the apoptosis and inflammation of pancreatic acinar AR42J cells induced by cerulein, a drug induced CP, or Conditioned Medium from PSCs (PSCs-CM) or the combination of PSCs-CM and cerulein. Moreover, SSd significantly inhibited the activated phosphorylation of JNK1/2, ERK1/2, and p38 MAPK induced by cerulein or the combination of PSCs-CM and cerulein in AR42J cells. Furthermore, SSd treatment markedly decreased the protein levels of p-JNK and p-p38 MAPK caused by PSCs-CM alone. In conclusion, SSd ameliorated pancreatic injury, suppressed AR42J inflammation and apoptosis induced by cerulein, interrupted the effect of PSCs-CM on AR42J cells inflammation and apoptosis, possibly through MAPK pathway.

## Introduction

Pancreatitis is an inflammatory disease of the pancreas and divided into acute and chronic pancreatitis. Chronic pancreatitis (CP) is characterized by irreversible inflammatory disorders that leading to fibrotic destruction of pancreatic parenchyma, endocrine, and exocrine dysfunction (Sheth et al., 2017). CP develops due to repeated episodes of acute pancreatitis (AP) resulting in the activation of pancreatic stellate cells (PSCs); thus fibrosis (Beyer et al., 2020). There is no specific treatment for CP. Elucidation to the new targets of the injured pancreas and the interplay with the cells will help identify potential therapies.

Previous studies on CP focuses on pancreatic stellate cells (PSCs), which majorly involved in the process of fibrosis. Acinar cell injury is the central to AP. However, the role of acinar cells in CP was less studied. It was well accepted that the process of cellular apoptosis of acinar cells was observed during the course of CP and the acinar cell apoptosis index was higher in CP than controls (Su et al., 2001; Bateman et al., 2002). In the early stage of CP, acinar cells were firstly damaged and initiated a cascade of pancreatic autodigestion and inflammation. The acinar cell injury involves acinar cell release of inflammatory mediators, activation of PSCs and recruitment of various immune cells (Patel Fine., 2005). The activated PSCs ultimately produce excessive extracellular matrix (ECM), manifested as pancreatic fibrosis (Chen et al., 2012; Erkan et al., 2012). In turn, the activated PSCs secret kinds of cytokines and chemokines that further facilitate the apoptosis of acinar cells (Xia et al., 2018). Therefore, preventing or suppressing the injury of acinar cells might be an efficacious therapeutic strategy for CP.

Matsushita et al. found that the apoptosis of pancreatic acinar cells was increased in a dibutyltin dichloride (DBTC) induced CP model (Matsushita et al., 2012). Apoptosis refers to a programmed form of cell death after being subjected to various stimuli (D’Arcy 2019). The role of the pro-apoptotic protein Bax and the anti-apoptotic protein Bcl-2 in regulating apoptosis are mainly to change the mitochondria Permeation of the outer membrane and regulate the release of cytochrome C. Then cytochrome C activates Caspase-9 and effector apoptotic protease Caspase-3. Afterwards, activated Caspase-3 trigger the cascade of apoptosis, and eventually lead to apoptosis (Jia et al., 2012). The MAPK family includes three members: p38 MAPK, c-Jun N-terminal kinase (JNK) and extracellular signal-regulated kinase 1 and 2 (ERK1/2). ERK, JNK, and p38 MAPK have been reported increased in mice CP model (Xu et al., 2018). Moreover, growing evidence demonstrated that the activation of MAPK cascades promoting cell apoptosis (Liu et al., 2018; Zakki et al., 2018).

Saikosaponin d (SSd) is a triterpenoid saponin compound derived from the plant *Bupleurum falcatum*. SSd has been reported to exert multiple pharmacological effects, including anti-inflammation activity (Wang et al., 2015). Recently, our previous study suggested that SSd inhibited autophagy of PSCs through PI3K/Akt/mTOR pathway, thus ameliorated pancreatic fibrosis (Cui et al., 2019), However, it is not clear whether SSd protect pancreatic injury of CP via regulating the apoptosis of pancreatic acinar cells.

In the present study, we measured the effects of SSd on DBTC induced pancreatic injury *in vivo*, and investigated the regulation of SSd on the cell apoptosis in AR42J cells *in vitro*. We found that SSd notably ameliorated pancreatic injury through reduced the number of apoptotic pancreatic acinar cells via inhibiting MAPK pathway. Furthermore, SSd protected the cerulein-induced injury of AR42J cells through suppressing MAPK pathway. Meanwhile, further exploration suggested that SSd interrupted the effects of PSCs-CM and the combination of PSCs-CM and cerulein on AR42J cells apoptosis through MAPK pathway. Thus, our findings might help to comprehensively elucidate the role of SSd in protecting pancreatic injury in CP.

## Methods

### Materials and Reagents

**FIGURE 1:**
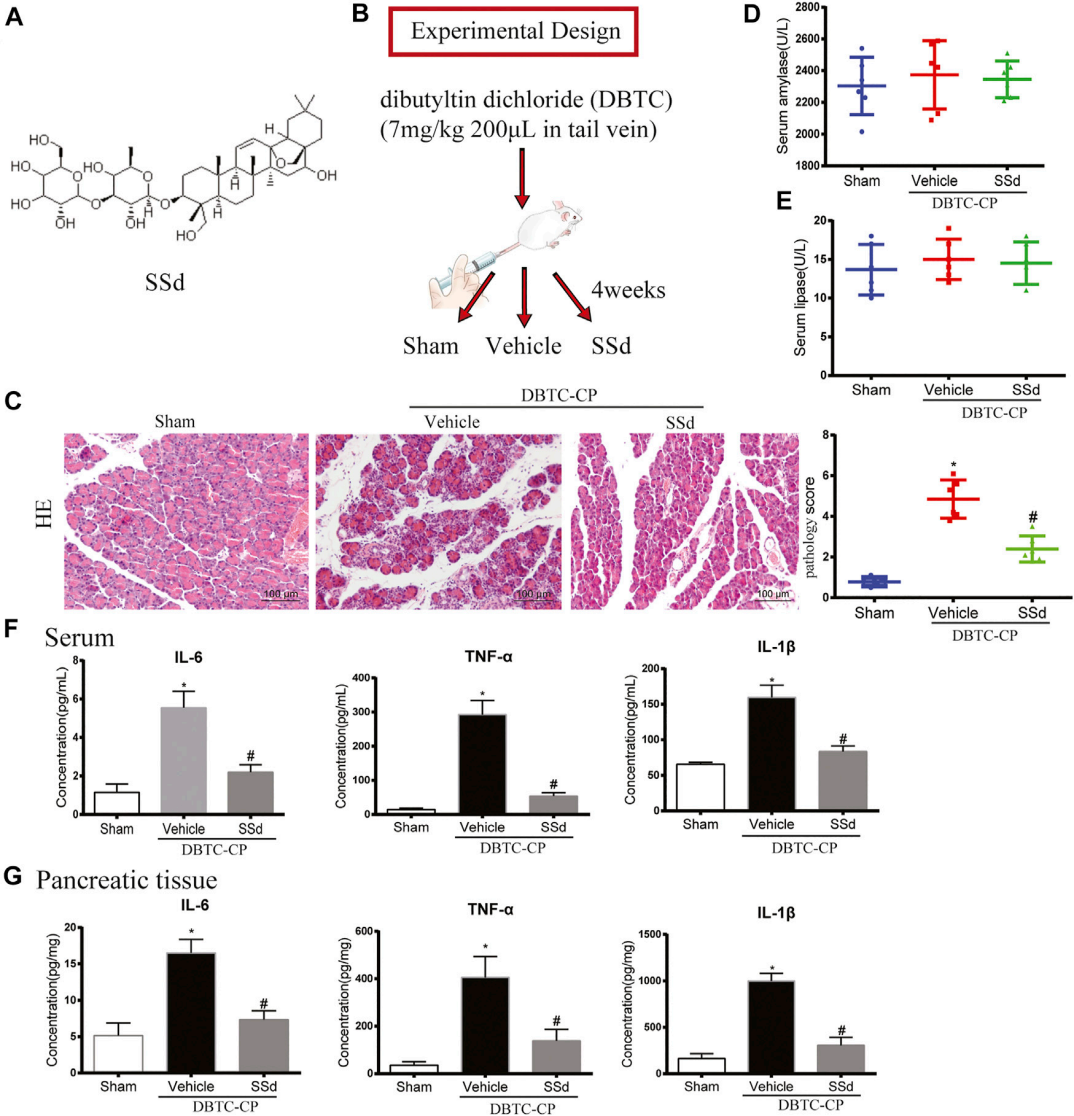
Effects of SSd on the histological changes, serum amylase and lipase in DBTC-induced CP **(A)** The chemical structure of SSd **(B)** Schematic of *in vivo* experiment design. **(C)** The pathological score of H and E staining (original magnification, ×200). **(D)** Serum amylase levels. **(E)** Serum lipase levels. **(F)** The levels of IL-6, TNF-α, and IL-1β in serum. **(G)** The levels of IL-6, TNF-α, and IL-1β in pancreatic tissue. (*n* = 6–8, ∗*p* < 0.05 vs. Sham group; #*p* < 0.05 vs. CP group).

Saikosaponin d (SSd) was purchased from Beijing Shenzhou Kechuang chemical technology research institute (purity>98%; Beijing, China). Its chemical structure is shown in the Figure 1A. GAPDH (#5174), Bax (#2772), Bcl-2 (#2876), Caspase-3 (#14220), Cleaved-caspase-3 (#9664), Caspase-9 (#9508), Phospho-p38 MAPK (#9211), p-JNK (#9255), JNK (#9252), and p-ERK1/2 (#8544) antibodies were obtained from Cell Signalling Technologies (Beverly, MA, United States). Cerulein (HY-A0190) were purchased from Med Chem Express (Shanghai, China).

### Animals and CP Model

Male Wistar rats (weighting, 150–170 g) were purchased from Beijing Huafukang Biotechnology Co., Ltd (Beijing, China). All experiments were conducted in accordance with the Chinese Guide for the Care and Use of Laboratory Animals as well as the methods for the management of experimental animals. All experiments were approved by the Medicine Ethical Committee of Tianjin Nankai hospital. All animals were randomly assigned into three groups including the control group, the CP group and the CP treated with SSd group (eight mice for each group) according to the experimental design outlined in Figure 1B.

The rat model of CP was induced by caudal vein injection of 7 mg/kg DBTC (Sigma Aldrich, United States) (Cui et al., 2019). Briefly, DBTC (Sigma-Aldrich, China) was dissolved in 100% ethanol first (one part), mixed with glycerol (two parts) and finally mixed with DMSO (two parts). The proportion of three solutions was 1:2:2. The CP group and SSd group were injected DBTC solution (7 mg/kg body weight) into the caudal vein. (Inoue et al., 2002). For the control group, the rats were injected with vehicle (ethanol:glycerol:DMSO, 1:2:2) only. The day after DBTC induction, the rats were orally administered with normal saline (with 0.5% carboxymethyl cellulose, (Na-CMC)) or 1 mg/kg/d SSd in 1.0 ml saline (with 0.5% Na-CMC). Rats were euthanized and analyzed after 4 weeks.

### Histological Examinations

Haematoxylin and eosin (H and E) staining was used to explore the changes of histology in the pancreas. Pancreas was rapidly removed and immediately fixed in 4% paraformaldehyde solution. Fixed tissues were cut into 5 μm sections and used for H and E staining. The pancreatic histopathology scores were calculated by evaluating the following criteria: edema, acinar vacuolization and necrosis, and proinflammatory cell infiltration, in accordance with previous reports (Niina, et al., 2014) (0 is normal and 4 is severe).

### Serum Parameters

Serum amylase and lipase activities were measured by using assay kits (C016-1, A054-2, Nanjing Jiancheng Bioengineering Institute, Nanjing, China) with spectrophotometric determination according to the manufacturer’s protocols.

### ELISA

The inflammatory factors IL-6, TNFα, and IL-1β in serum and pancreatic tissue were estimated by using assay kits (CSB-E04640r, CSB-E11987r, CSB-E08055r, CusaBio, Wuhan, China) according to the manufacturer’s protocols.

### *In Situ* TUNEL Fluorescence Staining Assay

A TdT-mediated dUTP nick-end labeling (TUNEL) assay (Promega, Wisconsin, United States) was performed to identify the apoptotic cell *in situ*. Briefly, the paraffined pancreatic sections were dewaxed to hydrate, covered with protease K for 30 min at 37°C. The slides were washed by PBS and then incubated with TUNEL reaction mixed solution at 37°C for 1 h. For analysis, the images were captured with fluorescence microscope (Leica, German) and the apoptotic cells were stained green. To avoid histological differences between the samples, five visual fields were randomly selected for each slice (six rats per group, *n* = 6). The Image Pro Plus 6.0 software were used to analyzed the percentage of TUNEL-positive cells (%) in the pancreas.

### Cell Lines and Culture Conditions

Rat pancreatic acinar AR42J cells were obtained from ProCell (CL-0025, Wuhan, Hubei, China) and cultured in Ham’s Fe12 K medium containing 20% fetal bovine serum and 1% penicillin/streptomycin.

### Cell Viability Assay

AR42J were seeded in 96-well plate with 5 × 10^3^ cells/well. Cells were treated with different concentrations of SSd (0, 1, 10, 20, 30, 40 or 50 μM) for 24 h, then added 10 μl Cell Counting Kit-8 solution (Beyotime, Shanghai, China) to each well. After incubation for another 4 h, the absorbance at 450 nm was determined by microplate reader. The percentage of viable cells was calculated using the following formula: Cell viability (%) = [(A450 treated-A450blank)/(A450 control-A450blank)] × 100.

### Isolation, Identification and Culture of Rat PSCs

The primary PSCs were isolated from rat pancreas using Nycodenz (Axis-Shield, Norway) density gradient centrifugation as reported previously (Li, et al., 2018). Then PSCs were cultured in DMEM (Gibco) supplemented with 10% fetal bovine serum and 1% antibiotics (penicillin/streptomycin). The third to fifth generation of PSCs were used for experiments.

### Preparation of Conditioned Medium From PSCs (PSCs-CM)

PSCs (passage 3) were seeded at 5 × 10^5^ cells per 10-cm plate in DMEM medium. PSCs reached 80% confluence and then placed in serum-free medium for 24 h. The medium was then collected for *in vitro* experiments.

### Reverse Transcription Quantitative Polymerase Chain Reaction (RT-qPCR)

Total RNA was isolated by Trizol (Takara, China) according to the manufacturer’s protocol. Total RNA (1 μg) of each sample was reverse-transcribed into cDNA using Revert Aid First Strand cDNA Synthesis Kit (K1622, Thermo, United States). qPCR was performed to quantify gene expression levels by using Go Taq^®^ Qpcr Master Mix Kit (cat.no. A6001, Promega, Wisconsin, United States). Primer sequences are listed in Table 1. The results were quantified as 2^-△△Ct^. The relative mRNA expression data are shown as the relative fold change normalized to GAPDH.

**TABLE 1:** Primers used for RT-qPCR analysis.

Primers	Forward sequence 5′-3′	Reverse sequence 5′-3′
Bax	TTG​CTA​CAG​GGT​TTC​ATC​CAG	TGT​TGT​TGT​CCA​GTT​CAT​CG
Bcl-2	GGG​GCT​ACG​AGT​GGG​ATA​CT	GAC​GGT​AGC​GAC​GAG​AGA​AG
IL-6	GTT​GCC​TTC​TTG​GGA​CTG​ATG	TAT​ATA​CTG​GTC​TGT​TGT​GGG​TGG​T
TNF-α	CTT​CTC​ATT​CCC​GCT​CGT​G	CAG​CTG​CTC​CTC​TGC​TTG​GTG​GTT​T
IL-1β	AGG​CTG​ACA​GAC​CCC​AAA​AG	CTC​CAC​GGG​CAA​GAC​ATA​GG
GAPDH	AGA​TGG​TGA​AGG​TCG​GTG​TG	CTG​GAA​GAT​GGT​GAT​GGG​TT

### Western Blot Analysis

Rat pancreas tissue samples and cell were lysed using Protein lysates RIPA buffer (Solarbio, Beijing, China). BCA protein assay Kit (Thermo, United States) was used to measure the protein concentrations. Protein samples were separated by SDS-PAGE and subsequently transferred to the PVDF membrane (Millipore, United States). After blocking with 5% non-fat milk, the membranes were probed with primary antibodies at 4°C overnight, followed by incubation with secondary antibodies (1:50,000) for 2 h at room temperature. The proteins were visualized using the Chemiluminescent Substrate Kit (Affinity Biosciences, United States) and calculated using the Chemidoc XRS System (Bio-Rad, United States).

### Apoptosis Analysis

Annexin V-FITC/PI apoptosis detection kit (Sungene Biotech, Tianjin, China) was used to detect the effect of SSd on AR42J apoptosis according to the manufacturer’s instruction. The samples were measured by a FACScan flow cytometer (Beckman, CA, United States) as previously described (Cui, et al., 2020).

### Statistical Analyses

Results were presented as Mean ± SD. Data were analyzed via one-way ANOVA (multiple groups) and GraphPad Prism software 6. p value <0.05 was considered as statistically significant.

## Results

### SSd Attenuates Pancreatic Injury in DBTC-Induced CP

In CP, due to the long-term injury of acinar cells resulting the atrophy of acinar cells and dysfunction of pancreas. To investigate the protective effect of SSd on pancreatic injury of CP, we comprehensively evaluated the pancreatic function of each group. As shown in Figure 1C, H and E staining indicated that SSd alleviated pancreatic injury in DBTC-induced CP rats. Pancreatic pathology score revealed that SSd effectively reduced pancreatic injury of CP (*p* < 0.05, Figure 1B). However, as shown in Figures 1D,E, the activities of serum amylase and lipase were no statistical significance in DBTC-induced rats. Additionally, we detected the expression of several key inflammatory factors in the CP model. We observed a gradual increase of the levels of IL-6, TNF-α, and IL-1β in serum and pancreatic tissues in DBTC-induced CP rats. Importantly, SSd treatment substantially decreased the expression of these inflammatory factors (Figures 1F,G).

### SSd Alleviates the Apoptosis of Pancreatic Cells in DBTC-Induced CP

**FIGURE 2:**
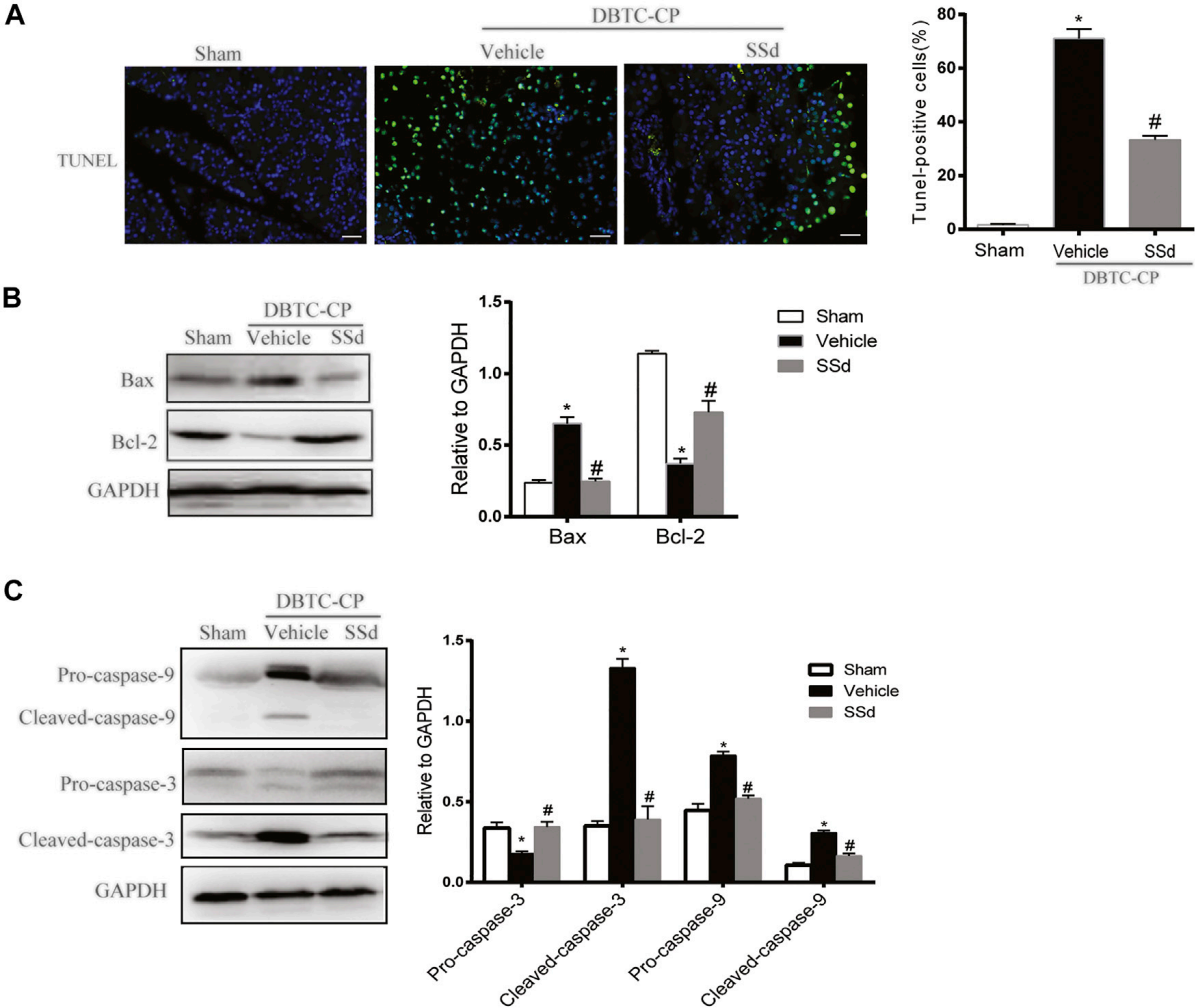
SSd alleviates pancreatic injury in DBTC-induced CP. **(A)** Apoptosis of pancreatic cells were detected by TUNEL staining. **(B)** Western blot analysis of BAX, Bcl-2 in the pancreas. **(C)** Western blot analysis of apoptotic proteins Pro-caspase-3, Cleaved-caspase-3, Pro-caspase-9, and Cleaved-caspase-9 in the pancreas. Data are shown as the mean ± SEM of at least three independent experiments. (*n* = 6–8, ∗*p* < 0.05 vs. Sham group; #*p* < 0.05 vs. CP group).

The development of CP was also accompanied by apoptosis and necrosis of pancreatic cells and it is a strategy to mitigate the pancreatic tissue damage by suppressing excessive apoptosis and necrosis (Erkan et al., 2012). TUNEL staining and western blot were used to investigate whether SSd can alleviate the apoptosis of pancreatic cells in CP rats. As shown in Figure 2A, the number of TUNEL-positive pancreatic cells was markedly elevated in DBTC-induced CP rats, While SSd notably decreased the number of TUNEL-positive pancreatic cells. Subsequently, western blot results suggested that the protein levels of pro-apoptotic protein Bax, cleaved caspase-9, and the cleaved caspase-3 were significantly increased whereas the expression of anti-apoptotic protein Bcl-2, Pro-caspase-9, and Pro-cspase-3 were reduced in DBTC-induced CP rats (*p* < 0.05). SSd treatment significantly inhibited the protein levels of Bax, Cleaved-caspase-9, and Cleaved-caspase-3, while increased the expression of Bcl-2, Pro-caspase-9 and Pro-caspase-3 (Figures 2B,C). These observations suggested that SSd administration suppressed the apoptosis of pancreatic cells caused by CP.

### SSd Treatment Suppressed MAPK Cascades in DBTC-Induced CP

**FIGURE 3:**
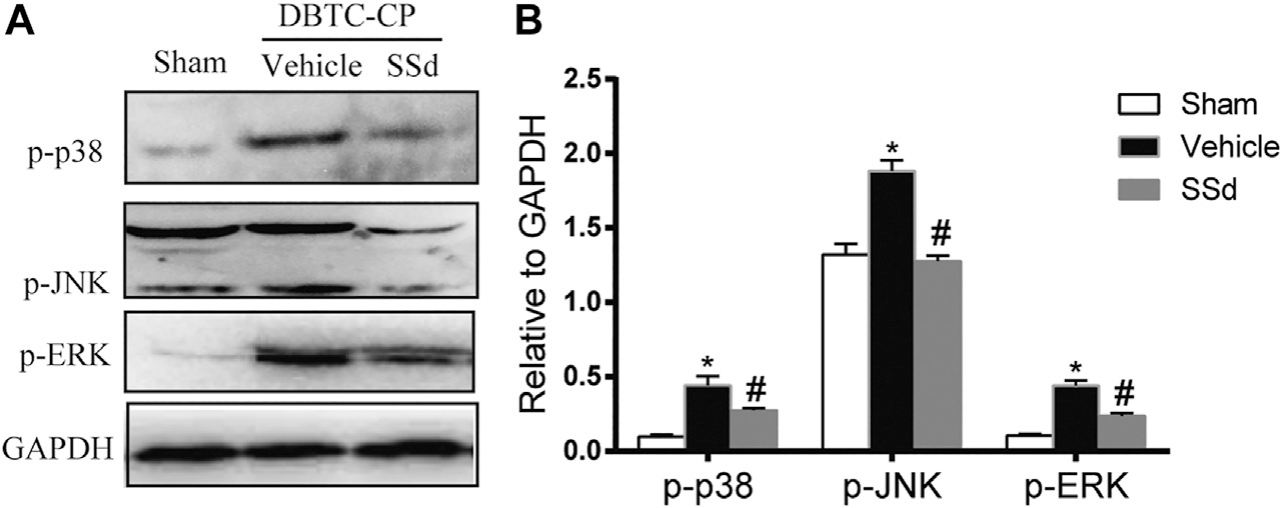
SSd inhibited the phosphorylation activation of MAPK family proteins in DBTC-induced CP. Western blot analysis of p-JNK, p-ERK 1/2, and p-p38 MAPK. (*n* = 6–8, ∗ *p* < 0.05 vs. Sham group; #*p* < 0.05 vs. CP group).

We analyzed the expression of the MAPK pathway in pancreatic tissues. The expression of p-JNK, p-ERK1/2, and p-p38 MAPK were significantly increased in the DBTC-induced CP group compared with the normal control group while SSd treatment notably decreased the levels of the p-JNK, p-ERK1/2 and p-p38 MAPK compared with the DBTC-induced CP group (Figure 3). Therefore, SSd could alleviate the pancreatic injury and inflammation *in vivo* maybe through suppressing the phosphorylation of MAPK pathway.

### SSd Attenuated Apoptosis and Inflammation of AR42J Cells Induced by Cerulein

**FIGURE 4:**
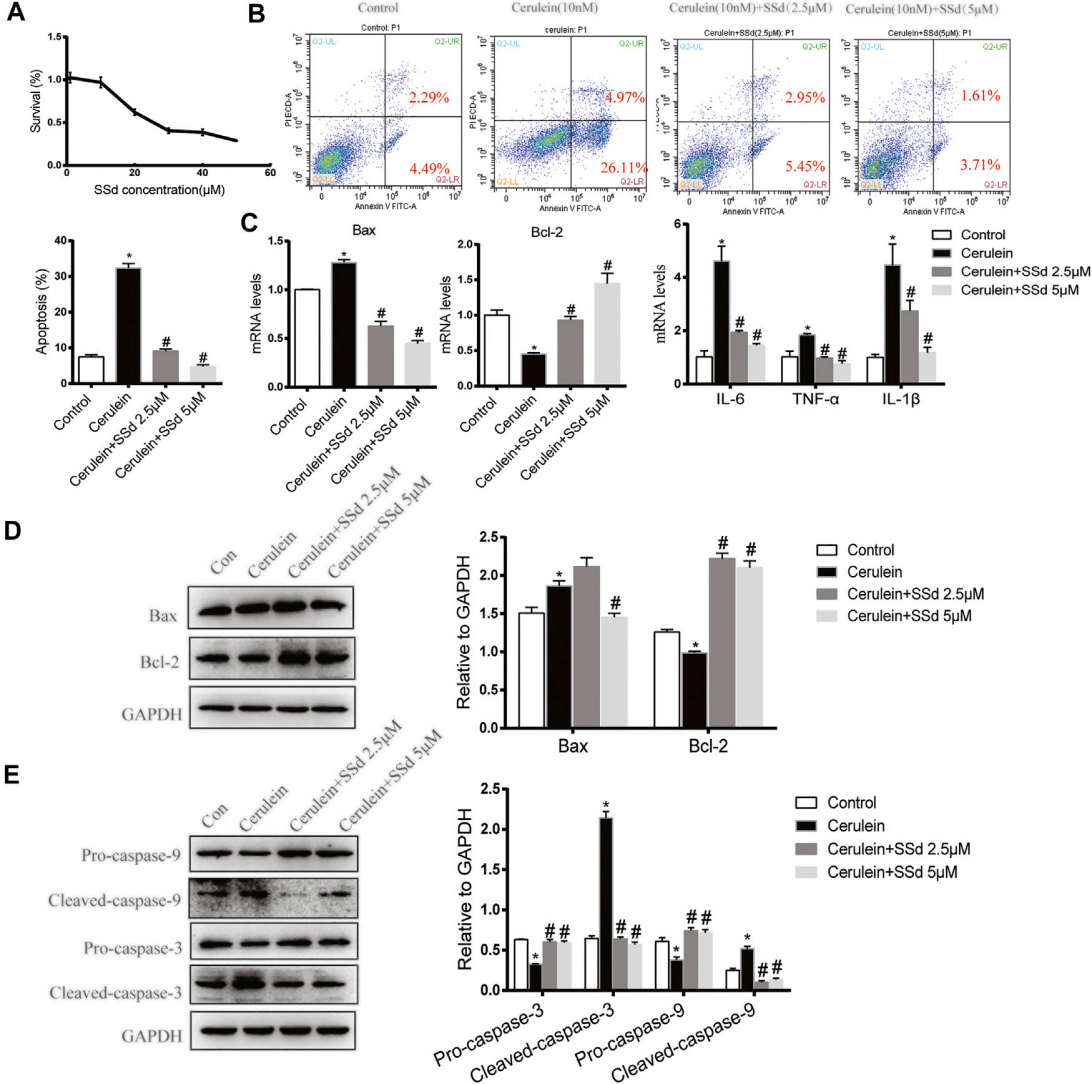
SSd attenuated apoptosis and inflammation of AR42J cells induced by cerulein. **(A)** CCK8 assay detected the cell viability of AR42J after SSd treatment. **(B)** Flow cytometry analysis of apoptosis of AR42J. AR42J were pre-treated with cerulein 10 nM for 1 h, then incubated with or without 2.5 μM or 5 μM SSd for 24 h. **(C)** RT-PCR analysis of Bax, Bcl-2, IL-6, TNF-α, and IL-1β. **(D)** Western blot analysis of Bax and Bcl-2. **(E)** Western blot analysis of C Pro-caspase-3, Cleaved-caspase-3, Pro-caspase-9, and Cleaved-caspase-9. Data are shown as the mean ± SEM of at least three independent experiments (*p < 0.05 compared with the control group, #*p* < 0.05 compared with the cerulein induced group).

Firstly, Cell viability of AR42J treated with different doses of SSd for 24 h was detected by CCK8. As shown in Figure 4A, the viability of AR42J gradually decreased in a dose-dependent manner with SSd administrated. Therefore, we choose 2.5 and 5 μM SSd (relevant cell viability was not affected) in following experiments. We next detected the protective role of SSd against cerulein-induced AR42J cells apoptosis, we measured cell apoptosis by staining the cells with Annexin V-FITC/PI. As shown in Figure 4B, cerulein (10 nM) significantly induced both early and late apoptosis of AR42J cells. Meanwhile, the mRNA transcription and protein levels of the Bax was significantly increased while Bcl-2 was markedly decreased induced by cerulein (Figure 4C). Western blot results indicated that cerulein significantly decreased the protein levels of the Pro-caspase-9 and the Pro-caspase-3 and increased the levels of the Cleaved-caspase-9 and the Cleaved-caspase-3 (Figures 4D,E). On the contrary, SSd treatment markedly suppressed the apoptotic events in AR42J cells pre-incubated with cerulein. Besides, we also found that SSd treatment notably decreased the levels of IL-6, TNF-α, and IL-1β in AR42J cells induced by cerulein (Figure 4C). Taken together, these findings suggested that SSd administration protected AR42J cells from apoptosis and inflammation induced by cerulein.

### SSd Reduced Apoptosis and Inflammation of AR42J Cells Induced by PSCs-CM or PSCs-CM and Cerulein Combination

**FIGURE 5:**
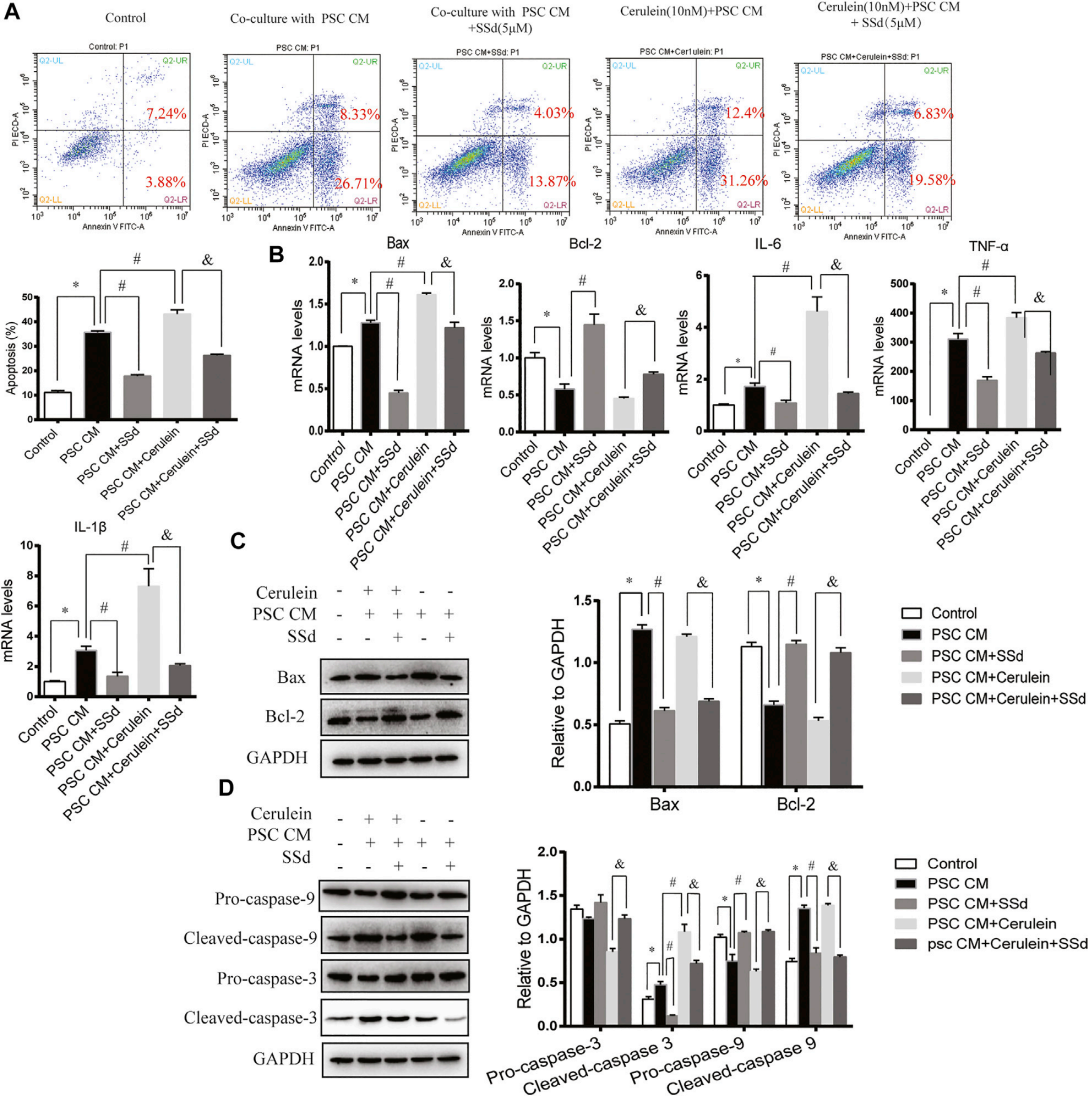
SSd reduced apoptosis and inflammation of AR42J cells induced by PSCs-CM or PSCs-CM and cerulein combination. AR42J cells were pre-treated with PSCs-CM with 5 μM SSd or 10 nM cerulein or 10 nM cerulein and 5 μM SSd for 24 h. **(A)** Flow cytometry analysis of apoptosis of AR42J. **(B)** RT-PCR analysis of Bax, Bcl-2, IL-6, TNF-α, and IL-1β. **(C)** Western blot analysis of Bax and Bcl-2. **(D)** Western blot analysis of Pro-caspase-3, Cleaved-caspase-3, Pro-caspase-9, and Cleaved-caspase-9. Data are shown as the mean ± SEM of at least three independent experiments. (*p < 0.05 compared with the control group, #*p* < 0.05 compared with the PSCs-CM group, and *p* < 0.05 compared with PSCs-CM + cerulein group).

In a previous study, Xia et al. found that the CM from LTC-14 PSCs instigated apoptosis in the AR42J cells (Xia, et al., 2018). In our co-culturing experiments, we detected the co-culturing effects of the CM from PSCs on the apoptosis and inflammation of AR42J cells. Consistent with previous finding, we found that the PSCs-CM could also induce the apoptosis and inflammation of AR42J. As shown in Figure 5A, PSCs-CM significantly promoted the apoptotic ratio of AR42J. PSCs-CM also markedly increased the mRNA levels of IL-6, TNF-α, IL-1β, and Bax while decreased the level of Bcl-2 (Figure 5B). Moreover, Western blotting result showed that the protein levels of Bax, Cleaved-caspase-3, and Cleaved-caspase-9 were significantly increased whereas the expression of Bcl-2 and Pro-caspase-9 decreased with PSCs-CM treatment (Figures 5C,D). But such detrimental effects of PSCs-CM were markedly declined by SSd treatment at 5 μM (Figures 5A–D).

Then, to analyze whether PSCs-CM and cerulein combination enhance the apoptosis of AR42J, AR42J was pretreated with cerulein for 1 h prior of PSCs-CM treatment. As expected, we observed that PSCs-CM and cerulein combination greatly induced the apoptosis ratio and the inflammation of AR42J compared with PSCs-CM treatment alone (Figures 5A,B). Additionally, PSCs-CM and cerulein combination substantially decreased the expression of Pro-caspase-3 while increased the protein level of Cleaved-caspase-3 compared with PSCs-CM treatment alone, but there was no significant difference in the Bax, Bcl-2, Pro-caspase-9, and Cleaved-caspase-9 between the PSCs-CM and cerulein combination group and the PSCs-CM treatment alone group. (Figures 5C,D). However, such apoptotic events were inhibited by the SSd administration at 5 μM (Figures 5A–D). In summary, these *in vitro* results showed that the inflammation and acinar cell apoptosis caused by PSCs-CM alone or PSCs-CM and cerulein combination were substantially supressed by SSd treatment.

### SSd Inhibited the Activity of MAPK Cascades in AR42J

**FIGURE 6:**
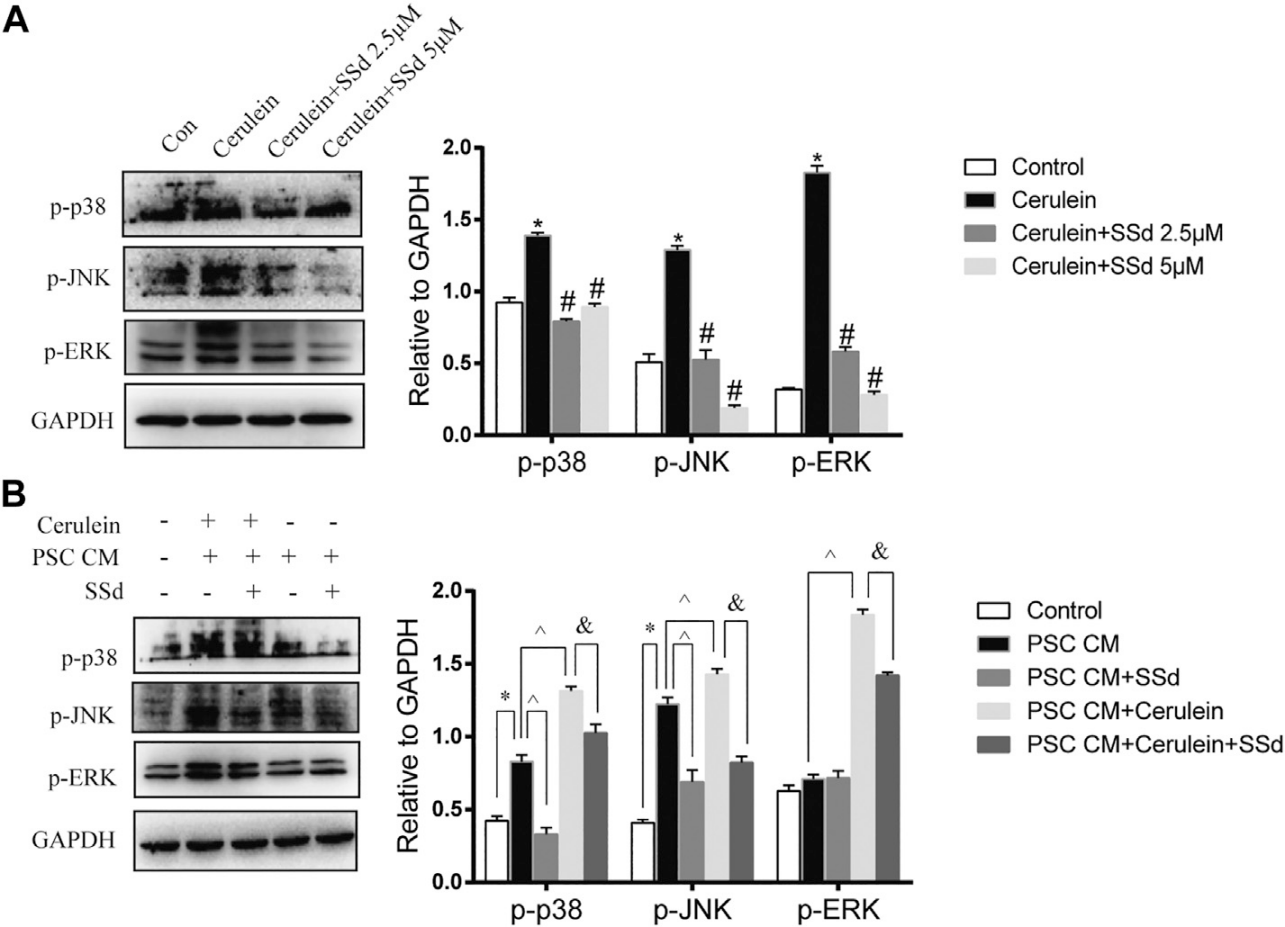
Effect of SSd on AR42J apoptosis and inflammation through the MAPK pathway. **(A)** Western blot analysis of p-JNK, p-ERK 1/2, and p-p38 MAPK. AR42J were pre-treated with cerulein 10 nM for 1h, then incubated with or without 5 μM or 10 μM SSd for 24 h. **(B)** Western blot analysis of p-JNK, p-ERK 1/2, and p-p38 MAPK. AR42J cells were pre-treated with PSCs-CM with 5 μM SSd or 10 nM cerulein or 10 nM cerulein and 5 μM SSd for 24 h. Data are shown as the mean ± SEM of at least three independent experiments. (*p < 0.05 compared with the control group, #*p* < 0.05 compared with the cerulein induced group, ^*p* < 0.05 compared with the PSCs-CM group, and *p* < 0.05 compared with PSCs-CM + cerulein group).

To further explore mechanism of the protective effects of SSd on the cerulein-induced apoptosis of AR42J, we detected the expression of MAPK pathway. As shown in Figure 6A, the increase in p-JNK, p-ERK1/2, and p-p38 MAPK expressions in AR42J cells induced by cerulein were greatly suppressed by SSd administration.

Moreover, the western blot results also showed that PSCs-CM significantly increased the expression of p-JNK and p-p38 MAPK, but had no effect on the expression of p-ERK while SSd treatment markedly decreased the protein levels of p-JNK and p-p38 MAPK (Figure 6B). Additionally, PSCs-CM and cerulein combination notably increased the phosphorylation of JNK1/2, ERK1/2, and p38 MAPK compared with PSCs-CM treatment alone, and SSd could significantly inhibit their phosphorylation levels (Figure 6B). These data demonstrated that the protection of SSd on the apoptosis and inflammation of AR42J cells is through down-regulated the phosphorylation of JNK1/2, ERK1/2, and p38 MAPK. Although exact molecular mechanisms of such an interplay remain to be investigated.

## Discussion

In the present study, to further confirm whether SSd could alleviate pancreatic injury during the development of CP, we using a DBTC-induced CP model. The results revealed that SSd attenuate pancreatic damage, decrease the apoptosis and suppress the phosphorylation level of MAPK family proteins (JNK1/2, ERK1/2, and p38 MAPK) significantly in the pancreas of CP rats. The *in vitro* studies have shown that SSd attenuated the apoptosis of AR42J induced by cerulein or PSCs-CM or the combination of PSCs-CM and cerulein through significantly inhibited the activated phosphorylation of JNK1/2 and p38 MAPK.

CP is characterized by inflammation and fibrosis. The injury of pancreatic acinar cells caused the inflammation and the excessive activation of PSCs induced the fibrosis. Recently, our previous study demonstrated that SSd inhibited autophagy of PSCs, thus ameliorated pancreatic fibrosis (Cui et al., 2019). The current study is the first study to investigate the effects of SSd treatment on the pancreatic injury and inflammation of CP via regulating the apoptosis of pancreatic acinar cells. Pancreatic acinar cells play a key role in the initiation of pancreatitis. Brigitte *et al.* reported that during the development of DBTC-induced CP in rats, there were distinct parenchymal cell damage, which characterized by a significant increase in serum amylase and lipase activities during the early period upon DBTC exposure. however, the activities returned to normal levels at days 28 (Glawe et al., 2005). In the present study, we used a rat experimental DBTC-induced CP model and also found that the activities of serum amylase and lipase were no statistical significance in DBTC-induced rats at days 28. Furthermore, Norio et al. reported that the apoptotic index of pancreatic acinar cells in DBTC administered rats were significantly increased (Matsushita et al., 2012). In this study, the results indicated that DBTC strikingly increased the number of apoptotic pancreatic acinar cells and the Western blot results of apoptotic-related protein levels of Bax, Bcl-2, Pro-caspase-9, Cleaved-caspase-9, Pro-caspase-3, and Cleaved-caspase-3 further confirmed this. Whereas SSd administration significantly reduced the apoptosis of acinar cells. MAPKs regulate many cellular processes, including growth, differentiation, survival, and apoptosis, as well as cytokine production, and have been reported increased in mice CP model (Lee et al., 2003). Our study indicated that SSd markedly inhibited the phosphorylation of JNK, ERK1/2, and p38 MAPK in DBTC-induced CP model.

Previous studies revealed that cerulein-stimulated apoptosis of AR42J cells via up-regulation of IL-6 (Yu et al., 2005). It is worth mentioning that SSd possesses anti-inflammatory activity. SSd suppresses pro-inflammatory cytokines including TNF-α, IL-6 (Lu, et al., 2012). In this study, we treated AR42J cells with 10 nM cerulein for 24 h *in vitro*. Similar to these aforementioned findings, our results showed that cerulein increased cytokines expression (including IL-6,TNF-α and IL-1β) and the apoptosis of AR42J cells. Whereas SSd could protect the pancreatic acinar cells from injury not only by decreased the expressions of IL-6 and TNF-α but also inhibited the apoptosis of AR42J cells. An increasing evidence demonstrated that initial damage to the acinar cells induced the activation of PSCs, which can thereby possibly further aggravate the destruction and apoptosis of more acinar cells, generating a vicious circle (Gryshchenko et al., 2018; Xia et al., 2018). Therefore, we also evaluated the impact of SSd on the inflammation and apoptosis induced by PSCs-CM or the combination of PSCs-CM and cerulein in AR42J cells. The results indicated that the CM from primary PSCs, is similar to cerulein, could also increase the mRNA levels of IL-6,TNF-α and IL-1β, and promote the apoptosis of AR42J cells. Then, SSd treatment reduced the apoptosis of AR42J cells induced by PSCs-CM. Furthermore, PSCs-CM and cerulein combination greatly induced the apoptosis and inflammation of AR42J compared with PSCs-CM treatment alone, but such detrimental effects were also markedly reduced by SSd administration. However, we don’t know that how many pro-inflammatory cytokines are contained in PSCs-CM and which cytokines of PSCs-CM are essential for inducing the apoptosis of AR42J. Interestingly, activation of PSCs can occur by both autocrine and paracrine mechanisms. Among them, autocrine cytokines include IL-1, IL-6, interleukin-8 (IL-8), TNF-α, transforming growth factor-beta1 (TGF-β1), platelet derived growth factor (PDGF), monocyte chemoattractant protein-1 (MCP-1) and so on (Bynigeri et al., 2017). Several studies showed SSd decreased the expression of pro-inflammatory cytokines including IL-6, TNF-α, and IL-1β (Wang et al., 2015; Ma et al., 2015). Therefore, we speculated that SSd exerts its anti-inflammatory effect by neutralizing the proinflammatory cytokines IL-6, TNF-α, and IL-1β contained in PSCs-CM. We will perform a large-scale cytokine analysis (or transcriptome analysis) to seek the essential cytokines in our future work.

We further investigated molecules that regulate apoptosis and inflammation after treatment with SSd. Cerulein induces cytokine expression and apoptotic cell death may be regulated by MAPK in pancreatic acinar cells (Lee et al., 2003). Lin, et al. reported SSd reduces HO-induced PC12 cell apoptosis by blocking MAPK-dependent oxidative damage (Lin et al., 2016). In order to explore the mechanisms, we detected the expression of the phosphorylation of JNK, ERK1/2, and p38 MAPK upon SSd treatment *in vitro*. The results indicated that SSd could reduce cerulein induced AR42J apoptosis via suppressing the MAPK signaling pathways. Moreover, we also found that PSCs-CM could significantly increase the protein levels of p-p38 and p-JNK, but there was no obvious alteration of p-ERK1/2. Additionally, PSCs-CM and cerulein combination notably increased the phosphorylation of JNK1/2, ERK1/2, and p38 MAPK compared with PSCs-CM treatment alone. Our study also demonstrated SSd strikingly suppressed the phosphorylation of JNK and p38 MAPK in both PSCs-CM alone and the combination of PSCs-CM and cerulein treatment in AR42J cells. A diverse set of transcription factors implicated in MAPK-induced apoptosis have been identified and validated (Cuenda et al., 2007; Zeke et al., 2016). Activator protein 1 (AP-1) and p53 were regulated by JNK and p38, which result in increased expression of pro-apoptotic proteins and decreased expression of anti-apoptotic proteins (Wang et al., 2021; Lu et al., 2020) and have been reported to be elevated in the apoptosis of pancreatic acinar cells (Lee et al., 2003; Tan et al., 2020). Therefore, we suspect that SSd regulate the apoptosis of AR42J cells induced by cerulein or PSCs-CM through AP-1 or p53 pathway. We need to verify it in our future work.

Of course, there are still several limitations exist in this study. Although we have shown the protective role of SSd from the pancreatic injury in DBTC-induced rats CP, we only measured the effect of a single dose of SSd. The dose-response effect of SSd in experimental CP remains unknown. So, we will confirm the optimal dosage of SSd in our future works. More importantly, we only measure the effects of PSCs-CM on the apoptosis of AR42J cells, but the effects of AR42J cells on the activation and apoptosis of PSCs were not detected. Besides, detailed mechanism of SSd on the effects of a crosstalk between PSCs and AR42J cells were not fully explain, which deserves further investigation.

In conclusion, the current study systematically explored the role of SSd in alleviating pancreatic injury *in vivo* and *in vitro*. Mechanistically, SSd exerted a marked protective effect on the inflammation and apoptosis of AR42J cells induced by cerulein via MAPK pathway. Furthermore, SSd interrupted the effects of PSCs-CM alone and the combination of PSCs-CM and cerulein on AR42J cells inflammation and apoptosis through MAPK pathway. Collectively, these results suggested that SSd could be a potential therapeutic agent for the clinical management of CP.

## Data Availability

The raw data supporting the conclusions of this article will be made available by the authors, without undue reservation.
