# Supplementary figures and images for: Saikosaponin D Attenuates Pancreatic Injury Through Suppressing the Apoptosis of Acinar Cell via Modulation of the MAPK Signaling Pathway
Source: Front Pharmacol. 2021 Oct 21;12:735079. doi: 10.3389/fphar.2021.735079 (PMC8566544; doi:10.3389/fphar.2021.735079)

FIG 3


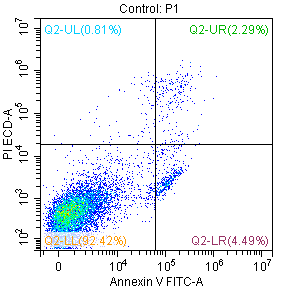

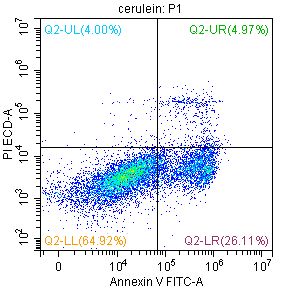


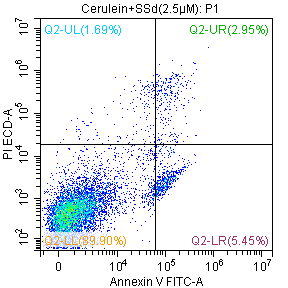


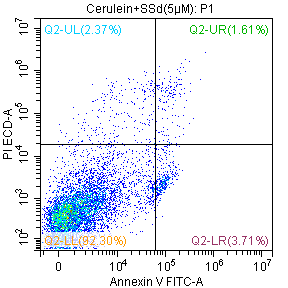

Supplement: Supplementary file 1 [file DataSheet1.ZIP › Flow cytometry data/Fig 4B apoptosis.docx]

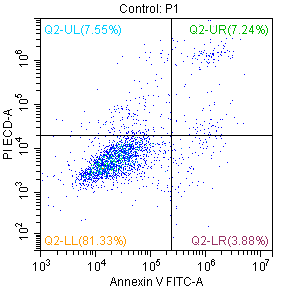


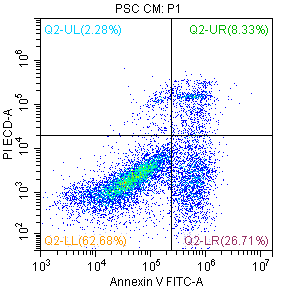


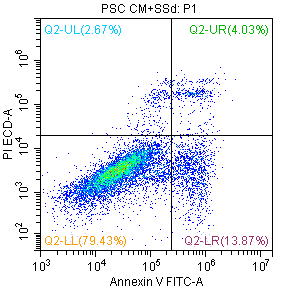

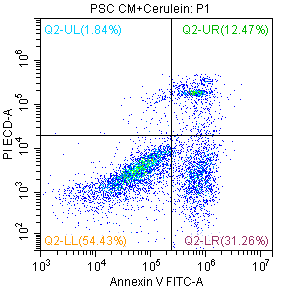


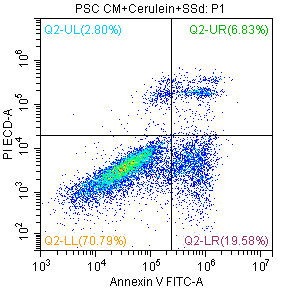

Supplement: Supplementary file 1 [file DataSheet1.ZIP › Flow cytometry data/Fig 5A apoptosis.docx]

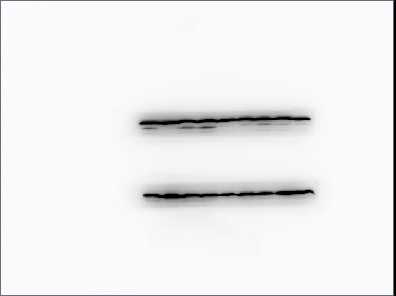

Supplement: Supplementary file 3 [file DataSheet2.ZIP › Original gels/Fig 2B-GAPDH.jpg]

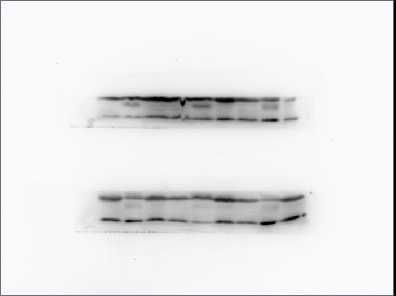

Supplement: Supplementary file 3 [file DataSheet2.ZIP › Original gels/Fig 2B-bax-1.jpg]

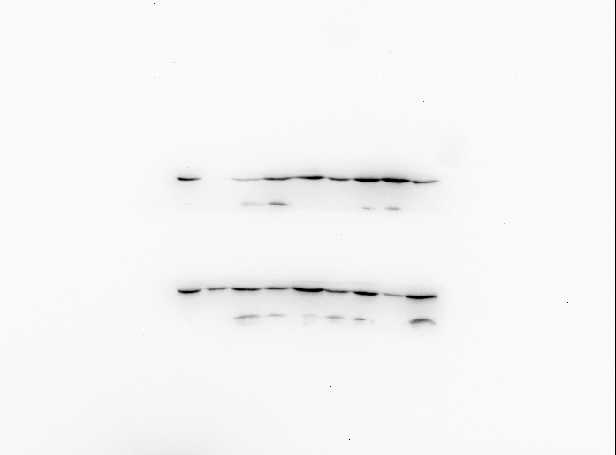

Supplement: Supplementary file 3 [file DataSheet2.ZIP › Original gels/Fig 2B-bcl-2.jpg]

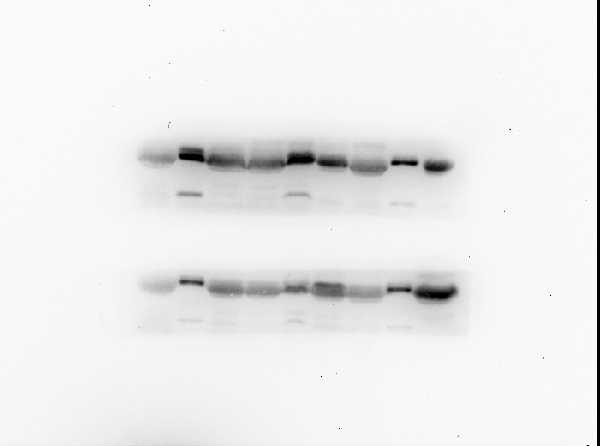

Supplement: Supplementary file 3 [file DataSheet2.ZIP › Original gels/Fig 2C-Caspase9.jpg]

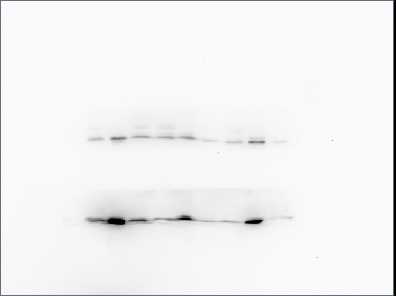

Supplement: Supplementary file 3 [file DataSheet2.ZIP › Original gels/Fig 2C-Cleaved-cas3.jpg]

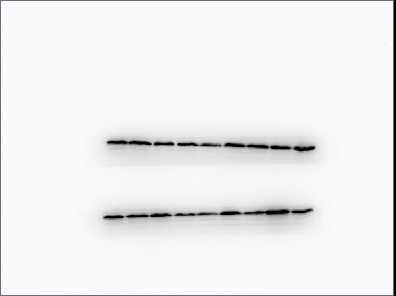

Supplement: Supplementary file 3 [file DataSheet2.ZIP › Original gels/Fig 2C-GAPDH.jpg]

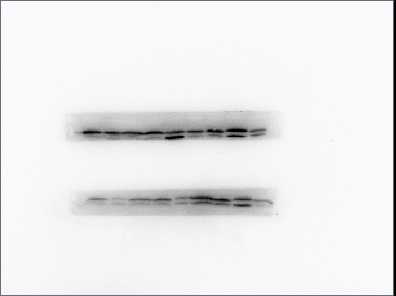

Supplement: Supplementary file 3 [file DataSheet2.ZIP › Original gels/Fig 2C-Pro-cas3.jpg]

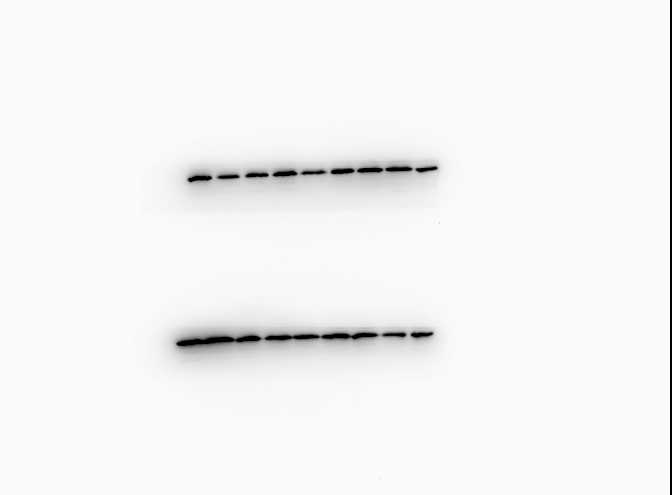

Supplement: Supplementary file 3 [file DataSheet2.ZIP › Original gels/Fig 3-GAPDH.jpg]

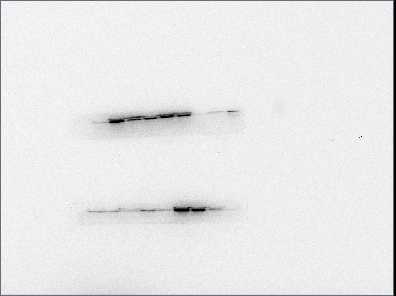

Supplement: Supplementary file 3 [file DataSheet2.ZIP › Original gels/Fig 3-p-ERK.jpg]

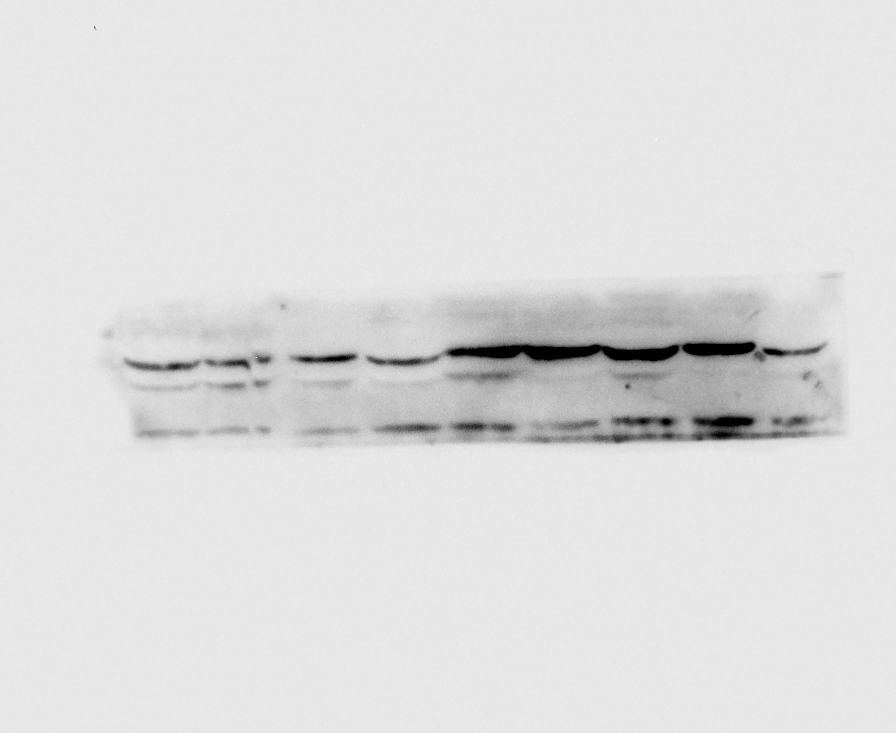

Supplement: Supplementary file 3 [file DataSheet2.ZIP › Original gels/Fig 3-p-JNK.tif]

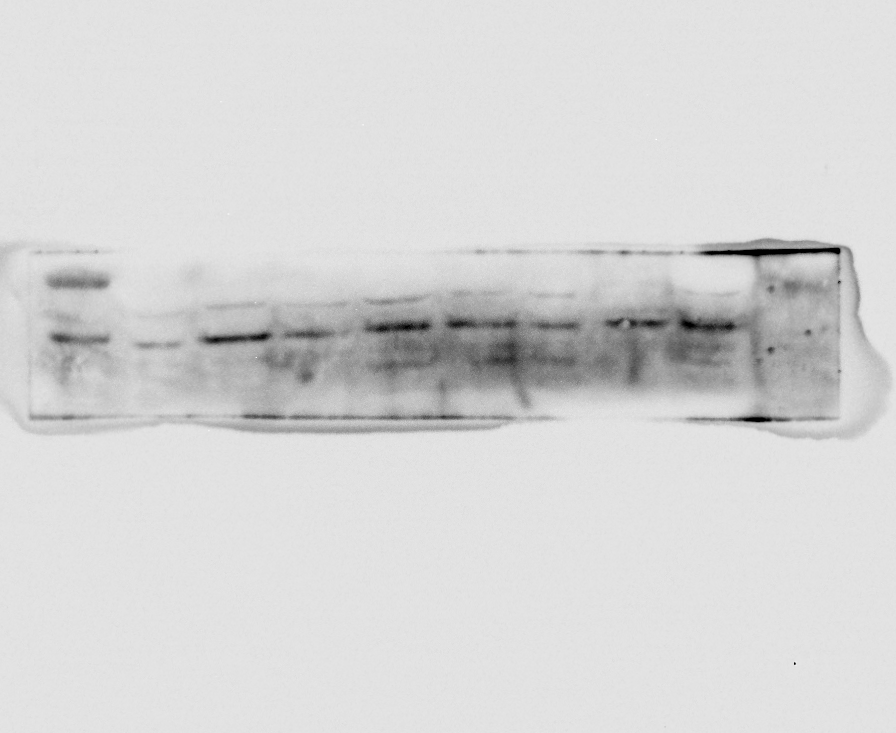

Supplement: Supplementary file 3 [file DataSheet2.ZIP › Original gels/Fig 3-p-p38-1.tif]

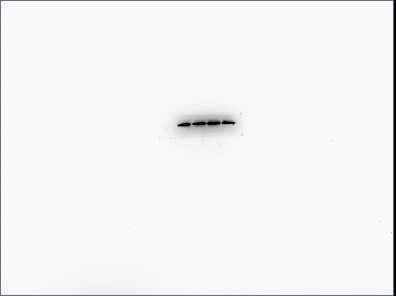

Supplement: Supplementary file 3 [file DataSheet2.ZIP › Original gels/Fig 4D-Bax.jpg]

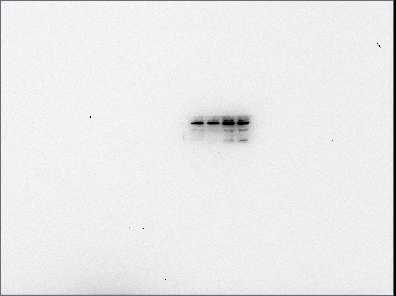

Supplement: Supplementary file 3 [file DataSheet2.ZIP › Original gels/Fig 4D-Bcl-2.jpg]

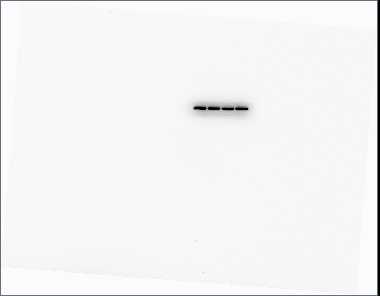

Supplement: Supplementary file 3 [file DataSheet2.ZIP › Original gels/Fig 4D-GAPDH.jpg]

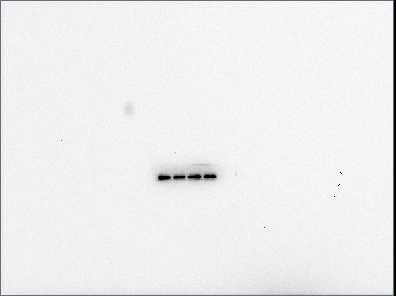

Supplement: Supplementary file 3 [file DataSheet2.ZIP › Original gels/Fig 4E Pro-caspase3.jpg]

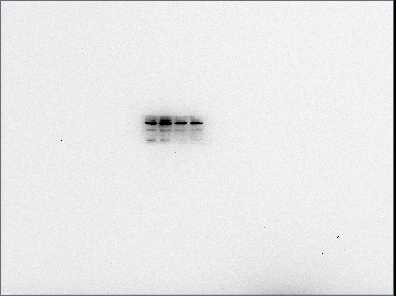

Supplement: Supplementary file 3 [file DataSheet2.ZIP › Original gels/Fig 4E-Cleaved-Caspase3.jpg]

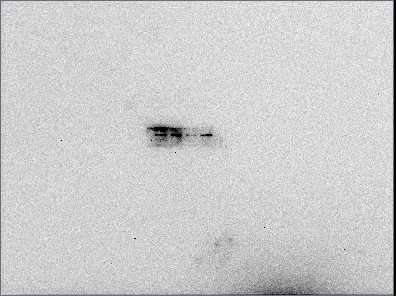

Supplement: Supplementary file 3 [file DataSheet2.ZIP › Original gels/Fig 4E-Cleaved-caspase9.jpg]

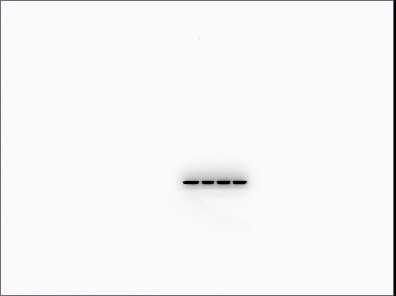

Supplement: Supplementary file 3 [file DataSheet2.ZIP › Original gels/Fig 4E-GAPDH.jpg]

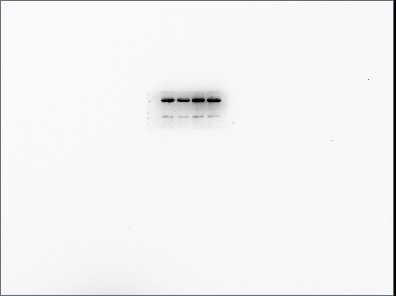

Supplement: Supplementary file 3 [file DataSheet2.ZIP › Original gels/Fig 4E-Pro-caspase9.jpg]

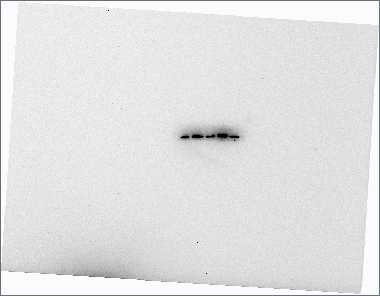

Supplement: Supplementary file 3 [file DataSheet2.ZIP › Original gels/Fig 5C-Bax.jpg]

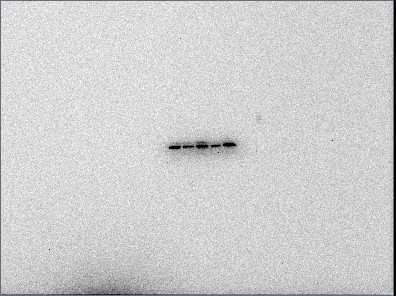

Supplement: Supplementary file 3 [file DataSheet2.ZIP › Original gels/Fig 5C-Bcl-2.jpg]

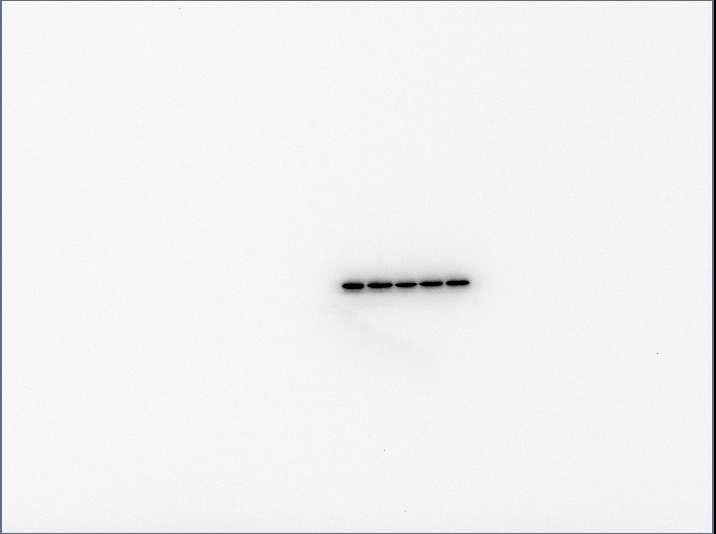

Supplement: Supplementary file 3 [file DataSheet2.ZIP › Original gels/Fig 5C-GAPDH.jpg]

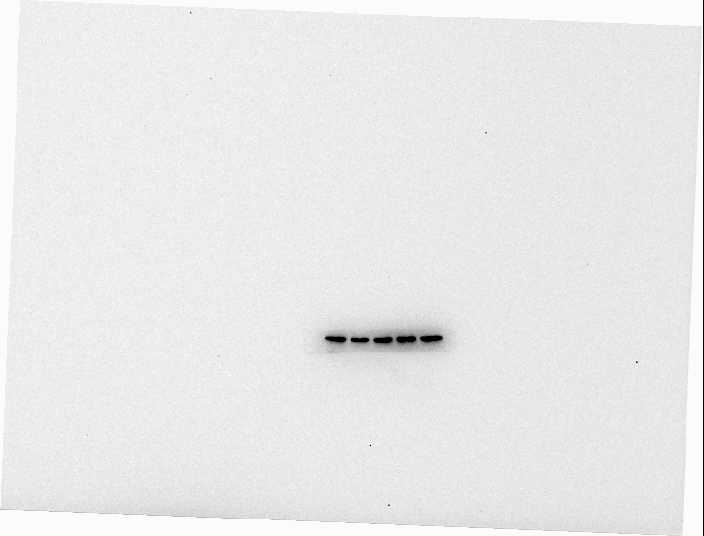

Supplement: Supplementary file 3 [file DataSheet2.ZIP › Original gels/Fig 5D Pro-caspase3 .jpg]

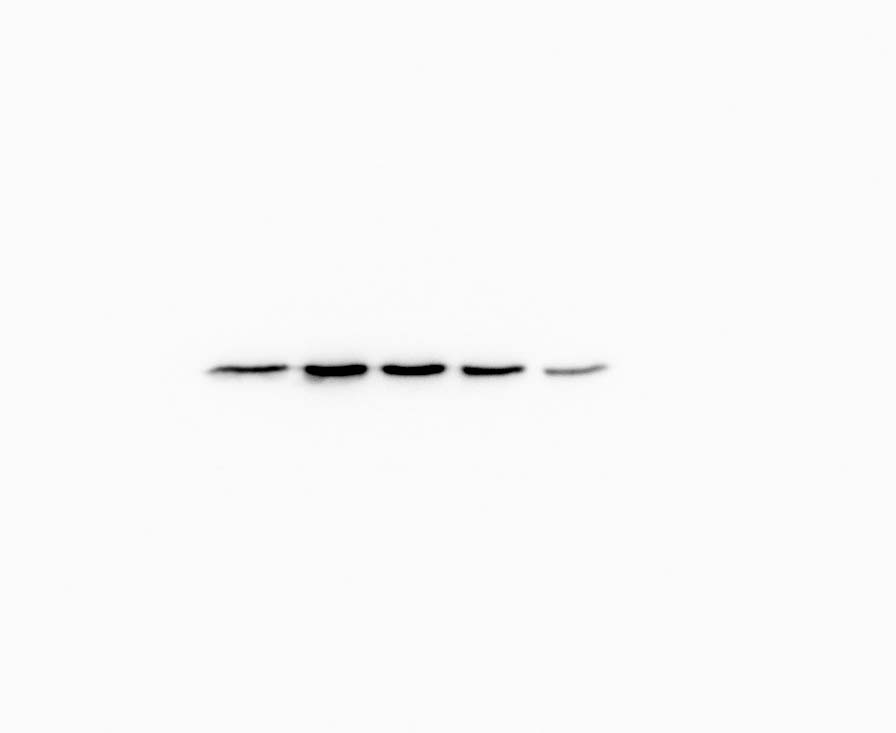

Supplement: Supplementary file 3 [file DataSheet2.ZIP › Original gels/Fig 5D-Cleaved-caspase3.jpg]

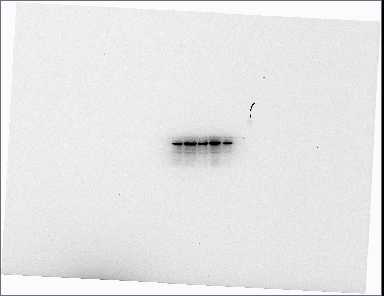

Supplement: Supplementary file 3 [file DataSheet2.ZIP › Original gels/Fig 5D-Cleaved-caspase9.jpg]

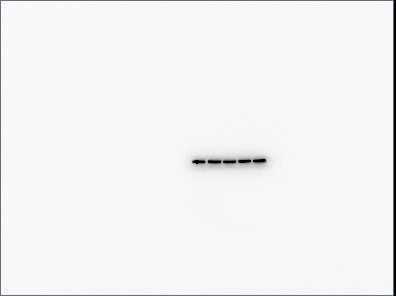

Supplement: Supplementary file 3 [file DataSheet2.ZIP › Original gels/Fig 5D-GAPDH.jpg]

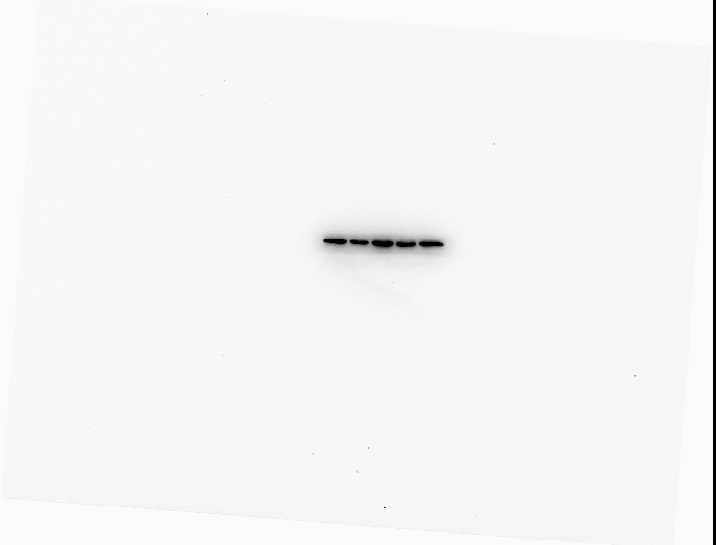

Supplement: Supplementary file 3 [file DataSheet2.ZIP › Original gels/Fig 5D-Pro-caspase 9.jpg]

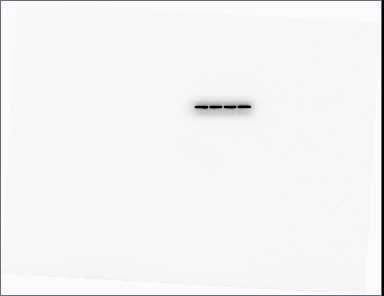

Supplement: Supplementary file 3 [file DataSheet2.ZIP › Original gels/Fig 6A-GAPDH.jpg]

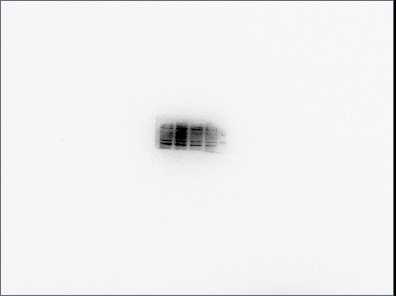

Supplement: Supplementary file 3 [file DataSheet2.ZIP › Original gels/Fig 6A-p-ERK.jpg]

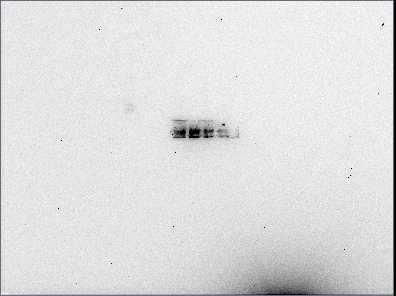

Supplement: Supplementary file 3 [file DataSheet2.ZIP › Original gels/Fig 6A-p-JNK.jpg]

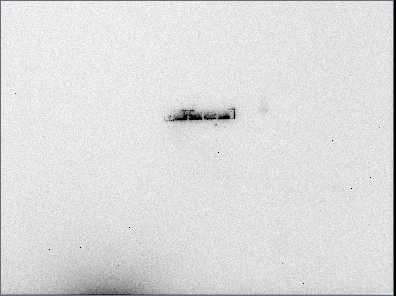

Supplement: Supplementary file 3 [file DataSheet2.ZIP › Original gels/Fig 6A-p-p38.jpg]

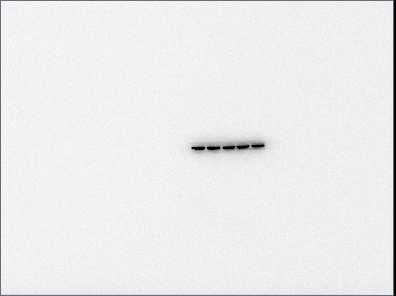

Supplement: Supplementary file 3 [file DataSheet2.ZIP › Original gels/Fig 6B-GAPDH.jpg]

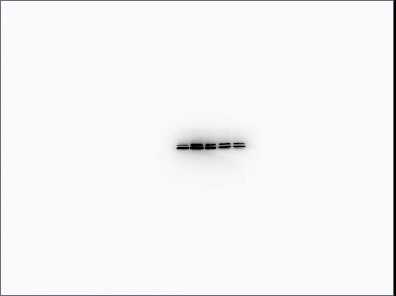

Supplement: Supplementary file 3 [file DataSheet2.ZIP › Original gels/Fig 6B-p-ERK.jpg]

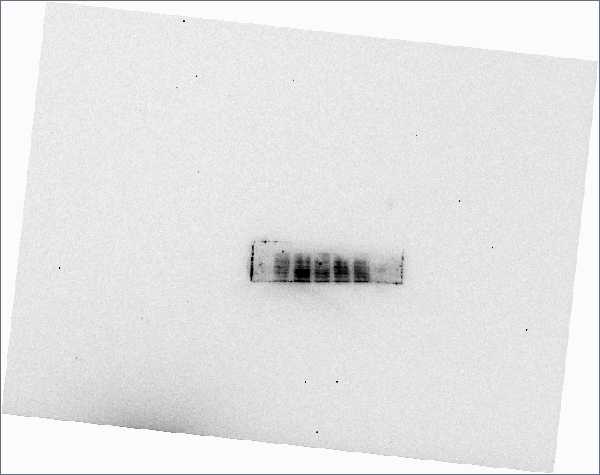

Supplement: Supplementary file 3 [file DataSheet2.ZIP › Original gels/Fig 6B-p-JNK.jpg]

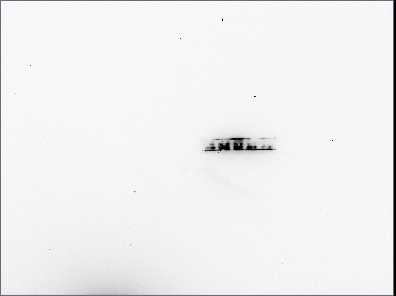

Supplement: Supplementary file 3 [file DataSheet2.ZIP › Original gels/Fig 6B-p-p38.jpg]
